# Neonatal intensive care unit admission among infants born full-term to CDC’s COVID-19 Vaccine Pregnancy Registry participants: A matched cohort study

**DOI:** 10.1080/21645515.2026.2636365

**Published:** 2026-03-03

**Authors:** Lauren Head Zauche, Sabrina A. Madni, David K. Shay, Christine K. Olson, Aliza Machefsky, Shana E. Godfred Cato, Andrea J. Sharma

**Affiliations:** a Immunization Safety Office, National Center for Emerging and Zoonotic Infectious Diseases, CDC, Atlanta, GA, USA; b U.S. Public Health Service Commissioned Corps, Rockville, MD, USA; c Department of Gynecology and Obstetrics, Emory University School of Medicine, Atlanta, GA, USA; d Division of Pediatric Emergency Medicine, University of Utah, Salt Lake City, UT, USA

**Keywords:** Neonatal intensive care unit, COVID-19 vaccine, maternal vaccination, pregnancy, vaccine safety

## Abstract

To estimate incidence of neonatal intensive care unit (NICU) admission among infants born to women receiving COVID-19 vaccines during pregnancy versus among infants born to unvaccinated women. We matched full-term infants from the United States Centers for Disease Control and Prevention’s (CDC) COVID-19 Vaccine Pregnancy Registry (C19VPR) to full-term infants from CDC’s Pregnancy Risk Assessment Monitoring System (PRAMS) with participant report of NICU admission available. We used 1:1 convenience sampling to match by maternal age, race, and ethnicity (n = 5,487 pairs). Adjusted incidence ratios (aIR) for NICU admission were calculated using Poisson regression; sensitivity analyses included a state-based match. NICU admission incidence was lower among C19VPR infants than PRAMS infants (7.7% vs 11.3%, aIR: 0.81, 95% CI: 0.65, 0.99). The highest aIR estimate generated through sensitivity analyses was 0.86 (95% CI: 0.67, 1.11). No evidence for an increased risk of NICU admission was found among infants born to vaccinated versus unvaccinated women.

## Introduction

Early in the COVID-19 pandemic (2020–2021), SARS-CoV-2 infection during pregnancy increased risks for neonatal morbidity and mortality, including admission to the neonatal intensive care unit (NICU) and neonatal SARS-CoV-2 infection.^1,2^ Data from the Surveillance for Emerging Threats to Mothers and Babies Network found that among neonates tested for SARS-CoV-2 born to women diagnosed with SARS-CoV-2 within 2 d of delivery, 4.6% were positive.^3^ Vaccination during pregnancy may confer protection to infants for the first months of life.^4,5^ No associations between COVID-19 vaccination and increased risk of spontaneous abortion^6^ or stillbirth^7^ have been documented. However, concerns about fetal health after COVID-19 vaccination may increase vaccine hesitancy.^8^

Data from the National Vital Statistics System show that 8.7–9.8% of all infants born in the United States during 2016–2023 were admitted to a NICU.^9^ NICU admission rates are lower for infants born term or post-term: early term (37–38 weeks’ gestation; 6.2–7.1%), full term (39–40 weeks’ gestation; 3.5–3.8%), and post-term (≥41 weeks’ gestation; 4.6–4.8%).^9^ Newborns are admitted to NICUs for many reasons.^10^ NICU admission has public health implications: it is associated with increased risk of infections, invasive medical interventions, increased family stress, and high costs to families.^10^ Several studies and meta-analyses found no increased risk of NICU admission among neonates born to COVID-19 vaccinated women compared to neonates of unvaccinated women.^11–17^ However, few studies have been conducted in the United States or have examined risk of NICU admission among full-term infants. Prematurity accounts for nearly half of NICU admissions in the United States.^10,18^ A substantial proportion (23.5–38%) of preterm deliveries are medically indicated due to maternal complications such as pre-eclampsia.^19,20^ An increased risk of preterm birth has not been observed among COVID-19 vaccinated pregnant populations.^11,13^ By assessing risk of NICU admission among full-term infants, the potentially confounding effect of preterm birth is eliminated.

The Centers for Disease Control and Prevention’s (CDC) COVID-19 Vaccine Pregnancy Registry (C19VPR) monitored pregnancy and infant outcomes following vaccination during or just prior to pregnancy to identify potential safety concerns.^21^ Our objective was to report incidence of NICU admission among infants in C19VPR. We estimated incidence of NICU admission among C19VPR participants’ infants born ≥37 weeks’ gestation and compared to a matched cohort of infants of unvaccinated women enrolled in CDC’s Pregnancy Risk Assessment Monitoring System (PRAMS).^22^

## Methods

### Study design and data sources

We conducted a matched cohort study using data from C19VPR and PRAMS. We identified potential participants for C19VPR among women ≥18 y who enrolled in CDC’s V-safe from December 2020 through June 2021.^21^ Participants resided in all 50 states, Washington D.C., and Puerto Rico. V-safe participants reporting receipt of ≥1 original monovalent COVID-19 vaccine (i.e., Pfizer-BioNTech, Moderna, or Johnson & Johnson/Janssen) in the 30 d before the pregnancy-associated last menstrual period or during pregnancy were invited to participate in C19VPR. Participant-reported data were collected about demographics, medical history, gestational health, pregnancy outcomes, delivery and postpartum complications, and infant health from January 2021 through August 2022. Complete C19VPR methodology is available.^21^

PRAMS is a cross-sectional state-based surveillance system used to monitor maternal and child health.^23^ Complete PRAMS methodology is available.^22^ Briefly, state birth certificate files were the sampling frame for identifying women who had a live birth during the surveillance year. Only one infant from multiple gestation pregnancies was eligible in each sample frame. Monthly, participating states drew a stratified random sample of 100–250 women 2–6 months after delivery. PRAMS sites oversampled subpopulations of interest based on research and programmatic needs. Because this was a matched cohort study, weighting of PRAMS data was unnecessary. The PRAMS questionnaire was mailed to women 2–6 months after a live birth to monitor pregnancy-related behaviors and experiences, with telephone follow-up for non-respondents. The PRAMS questionnaire varied by jurisdiction, consisting of core questions common to all jurisdictions, standard questions available for inclusion, and jurisdiction-developed questions.

To identify a cohort of PRAMS participants ≥18 y with no COVID-19 vaccination during pregnancy, we used data from 2019, 2020, and 2021. Participant report of NICU admission (“After your baby was delivered, was he or she put in an intensive care unit (NICU)?”) was available for inclusion in PRAMS questionnaires. To maximize comparability with C19VPR, participants from states implementing the NICU question formed the potentially eligible PRAMS comparison group; six states (Delaware, Kentucky, Mississippi, New Jersey, New Mexico, and Utah) included this question in 2019, 2020, and 2021 questionnaires. PRAMS participants from 2019 and 2020 were assumed to be unvaccinated and were eligible for matching as nearly all had given birth prior to large-scale administration of COVID-19 vaccines, which began December 14, 2020.^24^ Beginning in 2021, Delaware, New Jersey, and Utah included a question about COVID-19 vaccination in pregnancy. Women who reported not receiving a COVID-19 vaccine were eligible for matching. In total, 12,111 PRAMS infants were eligible for matching. C19VPR was reviewed by CDC, deemed not research, and was conducted consistent with applicable federal law and CDC policy, 45 C.F.R. part 46.102(l)(2), 21 C.F.R. part 56; 42 U.S.C. §241(d); 5 U.S.C. §552a; 44 U.S.C. §3501 et seq. The PRAMS protocol was approved by the CDC Institutional Review Board (IRB #2233).

### Study cohort and analyses

The full C19VPR cohort included 23,249 participants reporting on 23,628 fetuses or infants. We excluded non-live births (n = 2,251), preterm infants (n = 1,664), and infants missing a response to “Was your baby admitted to the neonatal intensive care unit?” (n = 160), resulting in 19,544 term live births.

Because the participant-report NICU admission question was implemented in only six states, we restricted the C19VPR cohort to participants from states with NICU admission rates in range of these six states (10.0% to 14.2%) based on 2016–2023 National Center for Health Statistics (NCHS) data,^9^ for the primary analysis (n = 7,547).^a^ Although NCHS data (birth certificate-based) are not directly comparable with participant-reported data, we assumed nondifferential misclassification of NICU admission across states.

We matched infants of C19VPR and PRAMS participants by maternal age categories (<30, 30–39, and ≥40 y) and maternal race and ethnicity (Non-Hispanic [NH] Black, NH-White, Hispanic, NH-Asian, and NH-Other). To maximize sample size, C19VPR infants from multiple gestation pregnancies (e.g., twins) were eligible to be matched individually; however, we were unable to match on plurality given limited sample size of multiple gestation pregnancies. We matched without replacement, as matching with replacement can reduce statistical independence, potentially biasing results.^25^ Several systematic imbalances between infants born prior to COVID-19 vaccine availability and those born while it was in use could exist; therefore, we preferentially matched C19VPR infants to PRAMS infants born in 2021. The primary analysis included 5,487 matched pairs.

**Figure 1.**
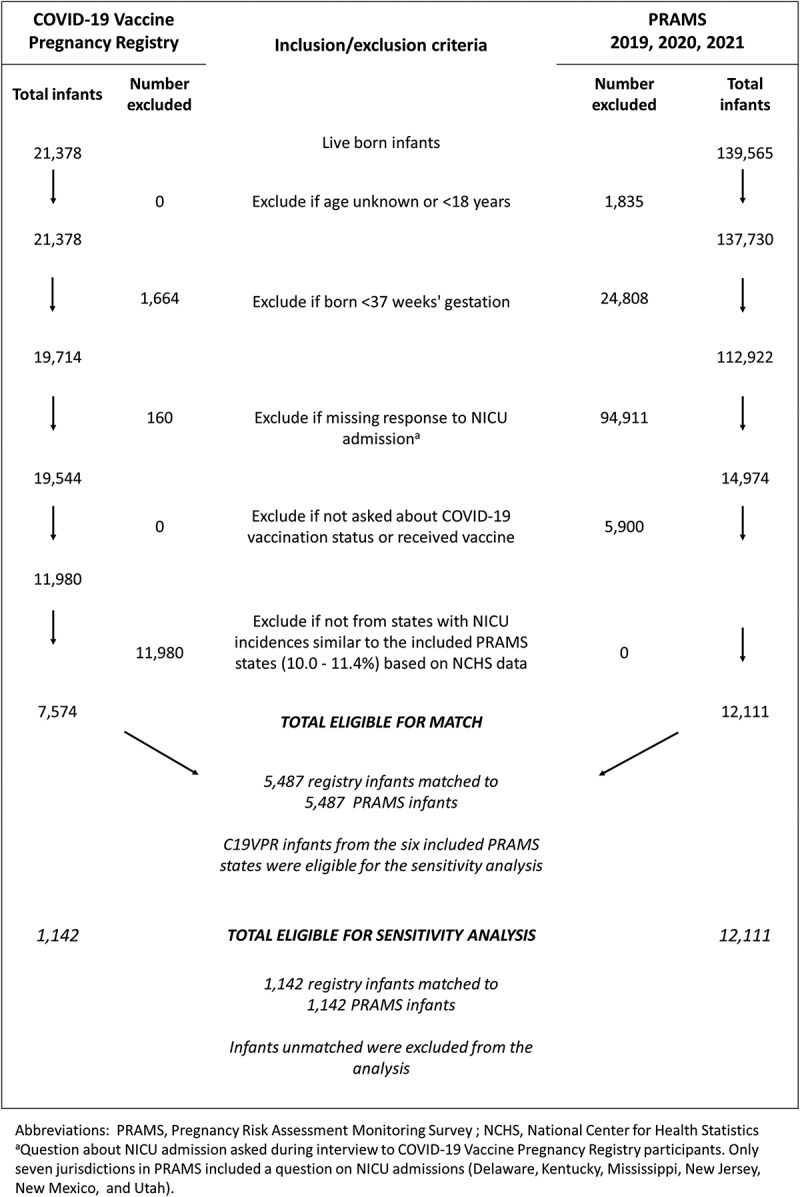
CDC COVID-19 vaccine pregnancy registry and pregnancy risk assessment monitoring systems consort diagram.

For a more direct comparison, we conducted a state-based sensitivity analysis that restricted the C19VPR cohort to infants in the six states that asked about NICU admission in PRAMS (n = 1,142 matched pairs; Figure 1). Because maternal COVID-19 illness during pregnancy was unknown for a large proportion of PRAMS participants, we conducted a sensitivity analysis for both the primary and state-based sensitivity analyses wherein we assumed all unknown COVID-19 illness statuses to be positive.

Descriptive statistics were calculated for matched participants and their infants for both C19VPR and PRAMS, and for unmatched participants and their infants from C19VPR. We assessed covariates associated with NICU admission in the C19VPR cohort including manufacturer of first COVID-19 vaccine dose received, timing relative to pregnancy of receipt of the COVID-19 vaccine dose conferring C19VPR eligibility (“registry-eligible vaccine”), maternal age group at delivery (<30, 30 to 39, or ≥40 y), race and ethnicity, healthcare personnel status, urbanicity of residence, parity, obesity (pre-pregnancy body mass index (BMI) ≥30 kg/m^2^), hypertension (preexisting or diagnosis of hypertensive disorder of pregnancy), diabetes (preexisting or gestational), plurality, delivery method, gestational age at delivery (early term, 37 to 38 weeks’ gestation; term, 39 to 40 weeks’ gestation; and late or post-term, ≥41 weeks’ gestation), infant birthweight category (<2,500, 2,500 to 3,999, and ≥4,000 grams), COVID-19 illness during pregnancy, and infant sex. We examined differences in delivery method (vaginal vs. cesarean) using a chi-square test to serve as a negative control as delivery method was not expected to be different based on vaccination status.

Poisson regression models with robust variance (accounting for matched pairs as clusters) were used to estimate crude and adjusted incidence ratios (aIR) and 95% confidence intervals (CIs) of NICU admission among C19VPR infants compared to PRAMS infants for both primary and sensitivity analyses.^26,27^ We used chi-squared tests to identify covariates associated with both COVID-19 vaccination and NICU admission. Covariates meeting this criterion and other covariates with theoretical importance were included in the multivariable model using a stepwise approach and retained if their inclusion resulted in a ≥ 10% change in the estimated association between exposure and outcome.^28,29^ Urbanicity, parity, hypertensive disorders of pregnancy, obesity, preexisting or gestational diabetes, COVID-19 illness during pregnancy, gestational age at delivery group, birthweight group, state, and birth year were identified as confounders. We found weak correlations (all Spearman r values < 0.20) among maternal confounders with known associations (i.e., obesity, hypertension, diabetes); therefore, these variables were included in the model. We found moderate correlation (Spearman r value = 0.22) between infant birthweight group and gestational age at delivery, so we excluded gestational age at delivery from models. Data about COVID-19 illness in pregnancy were collected for C19VPR participants but not for all PRAMS jurisdictions, so illness could not be included in comparative analyses.

SAS (version 9.4; SAS Institute, Cary, North Carolina, USA) was used for analyses.

## Results

Among infants born at term to women in the C19VPR cohort with information available about potential NICU admission (n = 19,544), most were born to participants who received either Pfizer-BioNTech (n = 11,563, 59.1%) or Moderna (n = 7,460, 38.2%) COVID-19 vaccines (Table 1). The registry-eligible vaccine was the first COVID-19 vaccine received for most (97.1%) participants. Mothers of about one-third of infants received a registry-eligible vaccine either within 30 d before their last menstrual period (n = 1,658, 8.5%) or during the first trimester (n = 5,502, 25.8%). Mothers of most infants were 30–39 y old (n = 1,282, 78.2%), NH White (n = 15,635, 80.0%), lived in urban areas (n = 18,295, 93.6%), and had a previous pregnancy (n = 11,433, 58.5%). A high percentage of infants had mothers who identified as healthcare personnel (n = 8,310, 42.5%). Most pregnancies were singletons (n = 19,235, 98.4%). Most infants were born vaginally (n = 13,841, 70.8%) and between 39–40 weeks’ gestation (n = 12,530, 64.1%).

**Table 1.** Bivariate associations between neonatal intensive care unit admission and covariates among full-term CDC COVID-19 Vaccine Pregnancy Registry infants (n = 19,544).^a^

			Neonatal intensive care unit (NICU) admission reported by participant				
	Total Infants		No		Yes		
	N = 19,544	Column%	n = 18,194	93.0%	n = 1,360	7.0%	*p*-value^b^
**Manufacturer of first COVID-19 vaccine dose**							.53
Pfizer-BioNTech	11,563	59.1	10,747	92.9	816	7.1	
Moderna	7,460	38.2	6,947	93.1	513	6.9	
Janssen	531	2.7	500	94.2	31	5.8	
**Timing of COVID-19 vaccine dose conferring C19VPR eligibility**							.76
Pre-pregnancy (up to 30 d before LMP)	1,658	8.5	1,533	92.5	125	7.5	
First trimester	5,052	25.8	4,709	93.2	343	6.8	
Second trimester	7,894	40.4	7,350	93.1	544	6.9	
Third trimester	4,950	25.3	4,602	93.0	348	7.0	
**Maternal age at delivery (years)**							.02
<30	3,436	17.6	3,180	92.6	256	7.5	
30–39	15,282	78.2	14,253	93.3	1,029	6.7	
40+	836	4.3	761	91.0	75	9.0	
**Maternal race/ethnicity**							.67
Non-Hispanic Black	416	2.1	385	92.6	31	7.5	
Non-Hispanic White	15,635	80.0	14,541	93.0	1,094	7.0	
Hispanic	1,788	9.1	1,662	93.0	126	7.0	
Non-Hispanic Asian	1,175	6.0	1,095	93.2	80	6.8	
Other	540	2.8	511	94.6	29	5.4	
**Healthcare personnel**							.05
No	11,001	56.3	10,206	92.8	795	7.2	
Yes	8,310	42.5	7,768	93.5	542	6.5	
Not reported	243	1.2	220	90.5	23	9.5	
**Urbanicity of residence**							
Urban	18,295	93.6	16,996	92.9	1,299	7.1	<.01
Rural	1,228	6.3	1,170	95.3	58	4.7	
Not reported	31	0.2	28	90.3	3	9.7	
**Nulliparous**							<.01
No	11,433	58.5	10,779	94.3	654	5.7	
Yes	8,121	41.5	7,415	91.3	706	8.7	
**Obesity**							<.01
No	15,752	80.6	14,743	93.6	1,009	6.4	
Yes	3,802	19.4	3,451	90.8	351	9.2	
**Hypertension (preexisting or HDP)**							<.01
No	16,696	85.4	15,645	93.7	1,051	6.3	
Yes	2,858	14.6	2,549	89.2	309	10.8	
**Diabetes Mellitus (preexisting or GDM)**							<.01
No	17,527	89.6	16,351	93.3	1,176	6.7	
Yes	2,027	10.4	1,843	90.9	184	9.1	
**SARS-CoV-2 infection in pregnancy**							.62
No	18,839	96.3	17,534	93.1	1,307	6.9	
Yes	715	3.7	662	92.6	53	7.4	
**Plurality**							<.01
Singleton	19,235	98.4	17,911	93.1	1,324	6.9	
Multiple	319	1.6	283	88.7	36	11.3	
**Delivery method**							<.01
Cesarean	5,679	29.0	5,045	88.8	634	11.2	
Vaginal	13,841	70.8	13,118	94.8	723	5.2	
Not reported	34	0.2	31	91.2	3	8.8	
**Birth year**							.31
2020	39	0.2	35	0.2	4	0.3	
2021	19,312	98.8	17,964	98.7	1,348	99.1	
2022	202	1.0	194	1.1	8	0.6	
Unknown	1	0.0	1	0.0	0	0.0	
**Gestational age at delivery**							<.01
Early term (37–38 weeks)	5,726	29.3	5,147	89.9	579	10.1	
Term (39–40 weeks)	12,530	64.1	11,849	94.6	681	5.4	
Late or post-term (≥41 weeks)	1,298	6.6	1,198	92.3	100	7.7	
**Birthweight (grams)**							<.01
<2500	338	1.7	271	86.5	67	19.8	
2500 to 3999	17,180	87.9	16,090	93.7	1,090	6.3	
≥4000	17,180	9.0	1,596	90.6	166	9.4	
Not reported	274	1.4	237	86.5	37	13.5	
**Infant sex**							<.01
Female	9,546	48.8	8,996	94.2	550	5.8	
Male	9,958	50.9	9,152	91.9	806	8.9	
Not reported	50	0.3	46	92.0	4	8.0	

Abbreviations: HPD, hypertensive disorder of pregnancy, GDM, gestational Diabetes Mellitus; LMP, last menstrual period.

^a^C19VPR enrolled women who were vaccinated just prior to or during pregnancy from December 15, 2020 through June 20, 2021. Infant birth dates ranged from December 19, 2020, to February 10, 2022.

^b^Chi Squared tests were used to assess the difference in distributions between groups.

NICU admission was reported for 1,360 (7.0%) full-term C19VPR infants. Manufacturer of COVID-19 vaccine, timing of COVID-19 vaccination relative to pregnancy (i.e., pre-pregnancy period or by trimester), and racial and ethnic group were not associated with NICU admission (Table 1). NICU admission was significantly associated with maternal age at delivery; participants aged 30–39 y reported NICU admission less often (6.7%) than younger (7.5%) and older participants (9.0%) (Table 1). Participants reporting NICU admission had a higher prevalence of obesity (9.2% vs 6.4%), hypertension (10.8% vs 6.3%), and diabetes (9.1% vs 6.7%) than those not reporting admission. First pregnancies (8.7% vs 5.7%), multiple gestation pregnancies (11.3% vs 6.9%), and cesarean deliveries (11.2% vs 5.2%) were associated with NICU admission. NICU incidence was higher among those born early-term (10.1%) and late or post-term (7.7%) compared to term (5.4%). A higher proportion of males were admitted to the NICU compared to females (8.9% vs 5.8%).

Incidence of NICU admission was higher among matched C19VPR infants versus unmatched (7.7% vs 6.7%, Table 2). Mothers of matched C19VPR infants were more likely to have received the Moderna vaccine (40.5% vs 37.2%). Mothers of all unmatched infants were over 30 y old, and 80.0% were NH-White. Timing of receipt of the registry-eligible vaccine was similar between matched and unmatched C19VPR participants.

**Table 2.** Characteristics of infants born to the CDC COVID-19 Vaccine Pregnancy Registry participants: matched vs. unmatched pregnant women (n = 19 554).^a^

	Unmatched		Matched		*p*-value^b^
	n = 14,067	Col %	n = 5,487	Col %	
**Neonatal intensive care unit admission**					<.01
No	13,131	93.4	5,063	92.3	
Yes	936	6.7	424	7.7	
**Manufacturer of first COVID-19 vaccine dose**					<.01
Pfizer-BioNTech	8,433	60.0	3,310	57.0	
Moderna	5,238	37.2	2,222	40.5	
Janssen	396	2.8	135	2.5	
**Timing of COVID-19 vaccine dose conferring C19VPR eligibility**					.97
Pre-pregnancy (up to 30 d before LMP)	1,190	8.5	468	8.5	
First trimester	3,631	25.8	1,421	25.9	
Second trimester	5,671	40.3	2,223	40.5	
Third trimester	3,575	25.4	1,375	25.1	
**Maternal age at delivery (years)**					<.01
<30	2,109	15.0	1,317	24.2	
30–39	11,364	80.8	3,918	71.4	
40+	594	4.2	242	4.4	
**Maternal race/ethnicity**					.04
Non-Hispanic Black	283	2.0	133	2.4	
Non-Hispanic White	11,255	80.0	4,380	79.8	
Hispanic	1,313	9.3	475	8.7	
Non-Hispanic Asian	850	6.0	325	5.9	
Other	366	2.6	174	3.2	
**Healthcare personnel**					<.01
No	8,053	57.3	2,948	53.7	
Yes	5,851	41.6	2,459	44.8	
Not reported	163	1.2	80	1.5	
**Urbanicity of residence**					<.01
Urban	13,215	93.9	5,080	92.6	
Rural	821	5.8	407	7.4	
Not reported	31	0.2	0	0.0	
**Nulliparous**					.04
No	8,289	58.9	3,144	57.3	
Yes	5,778	41.1	2,343	42.7	
**Obesity**					.94
No	11,330	80.5	4,422	80.6	
Yes	2,737	19.5	1,065	19.4	
**Hypertension (preexisting or HDP)**					.75
No	12,004	85.3	4,692	85.5	
Yes	2,063	14.7	795	14.5	
**Diabetes Mellitus (preexisting or GDM)**					.11
No	12,578	89.4	4,949	90.2	
Yes	1,489	10.6	538	9.8	
**SARS-CoV-2 infection in pregnancy**					.48
No	13,561	96.4	5,278	96.2	
Yes	506	3.6	209	3.8	
Unknown	1	0.0	0	0.0	
**Plurality**					.41
Singleton	13,831	98.3	5,404	98.5	
Multiple	236	1.7	83	1.5	
**Delivery method**					.96
Cesarean	4,092	29.1	1,587	28.9	
Vaginal	9,951	70.7	3,890	70.9	
Not reported	24	0.2	10	0.2	
**Birth year**					<.01
2020	27	0.2	12	0.2	
2021	13,872	98.6	5,440	99.1	
2022	167	1.2	35	0.6	
Not reported	1	0.0	0.0	0.0	
**Gestational age at delivery**					<.01
Early term (37–38 weeks)	4,104	29.2	1,622	29.6	
Term (39–40 weeks)	8,981	63.8	3,549	64.7	
Late or post-term (≥41 weeks)	982	7.0	316	5.8	
**Birthweight (grams)**					.22
<2500	233	1.7	105	1.9	
2500 to 3999	12,339	87.7	4,841	88.2	
≥4000	1,299	9.2	463	8.4	
Not reported	196	1.4	78	1.4	
**Infant sex**					.08
Female	7,153	50.9	2,805	51.1	
Male	6,871	48.8	2,675	48.8	
Not reported	43	0.3	7	0.1	

Abbreviations: HPD, hypertensive disorder of pregnancy, GDM, gestational Diabetes Mellitus; LMP, last menstrual period.

^a^C19VPR enrolled women who were vaccinated just prior to or during pregnancy from December 15, 2020 through June 20, 2021. C19VPR infant birth dates ranged from December 19, 2020 to February 10, 2022. PRAMS participants were enrolled from 2019–2021; infants in PRAMS were born during 2019–2022.

^b^Chi Squared tests were used to assess the difference in distributions between matched and unmatched groups. Missing values were excluded from chi-squared tests assessing distribution for delivery method and infant sex.

**Figure 2.**
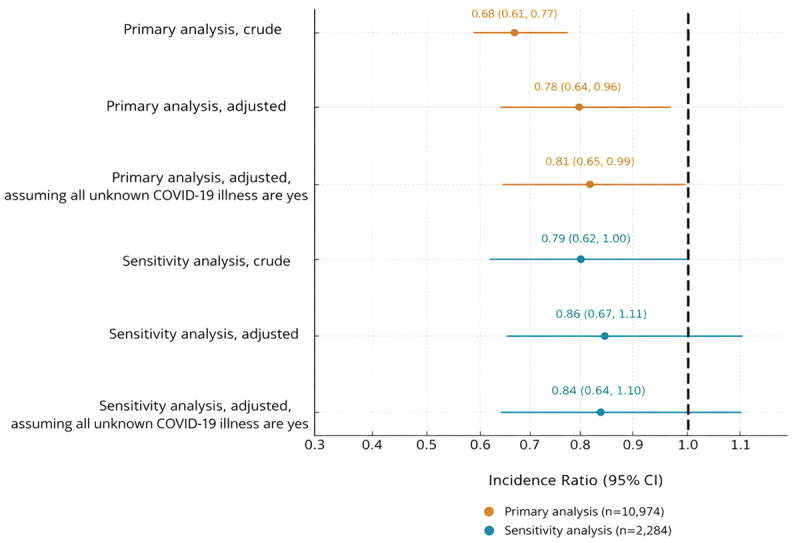
Adjusted^a^ incidence ratio of neonatal intensive care unit (NICU) admission among full-term infants in CDC’s COVID-19 Vaccine Pregnancy Registry (C19VPR)^b^ compared with the pregnancy risk Assessment Monitoring System (PRAMS) based on participant report.^c^

Table 3 displays characteristics of PRAMS and C19VPR infants in the primary analysis. Compared to C19VPR participants, more PRAMS participants had obesity, hypertension, and diabetes, and fewer PRAMS participants lived in urban areas and were nulliparous. More C19VPR participants reported known SARS-CoV-2 infection during pregnancy compared to PRAMS participants. More infants were born early-term and late or post-term among C19VPR. More infants were born <2,500 grams or ≥4,000 grams in PRAMS. There were no significant differences for plurality or infant sex. We found no differences between C19VPR and PRAMS participants for delivery method (*p* = .96 [Table 3]). After adjusting for confounding, participant-reported NICU admission was lower among C19VPR infants compared to PRAMS infants (7.7 vs 11.3%, aIR: 0.81, 95% CI: 0.65, 0.99). Results were consistent in the state-based sensitivity analysis and in analyses assuming all women with unknown COVID-19 illness status during pregnancy did have COVID-19 illness (Figure 2). The highest point estimate was observed in the adjusted sensitivity analysis (aIR: 0.86, 95% CI: 0.67, 1.11).

Primary analysis: Infants born to women participating in C19VPR from states with similar NICU admission rates to included PRAMS states based on NCHS data (10.0–11.2%; Alaska, Alabama, Arkansas, Colorado, District of Columbia, Delaware, Iowa, Idaho, Illinois, Indiana, Kentucky, Louisiana, Missouri, Mississippi, Montana, North Dakota, Nebraska, New Jersey, New Mexico, Nevada, New York, Ohio, Pennsylvania, South Carolina, South Dakota, and Utah).

Sensitivity analysis: Infants born to women in C19VPR from included PRAMS states (Delaware, Kentucky, Mississippi, New Jersey, New Mexico, and Utah).

^a^Adjusted for urbanicity, parity, hypertensive disorders of pregnancy, high body mass index (≥30 kg/m^2^), preexisting or gestational diabetes, COVID-19 illness during pregnancy, gestational age at delivery group (37–38 weeks’ gestation, 39–40 weeks’ gestation, and ≥41 weeks’ gestation), birthweight group (<2,500 grams, 2500–3999 grams, and ≥4,000 grams), state, and birth year.

^b^C19VPR enrolled women who were vaccinated just prior to or during pregnancy from December 15, 2020 through June 20, 2021. Infant birth dates ranged from December 19, 2020 to February 10, 2022.

^c^PRAMS participants include those from six states with participant report of NICU admission. Participants from 2021 and 2022 were excluded if they reported receiving a COVID-19 vaccine or if they did not provide a response to question asking whether they received a COVID-19 vaccine.

**Table 3.** Demographic characteristics of CDC COVID-19 Vaccine Pregnancy Registry (C19VPR) infants and infants born to Pregnancy Risk and Monitoring System (PRAMS) participants from 2019–2022 matched on maternal race and ethnicity and age.

	PRAMS 2019–2022^a^ (unvaccinated)		C19VPR 2020–2022^b^ (vaccinated)		*p*-value^c^
	n = 5,487	Col %	n = 5,487	Col %	
**Participant report of neonatal intensive care unit admission**					<.01
No	4,865	88.7	5,063	92.3	
Yes	622	11.3	424	7.7	
**Manufacturer of first COVID-19 vaccine dose**					N/A
Pfizer-BioNTech	–	–	3,310	57.0	
Moderna	–	–	2,222	40.5	
Janssen	–	–	135	2.5	
**Timing of COVID-19 vaccine dose conferring C19VPR eligibility**					N/A
Pre-pregnancy (≤30 d before LMP)	–	–	468	8.5	
First trimester	–	–	1,421	25.9	
Second trimester	–	–	2,223	40.5	
Third trimester	–	–	1,375	25.1	
**Maternal age at delivery (years)**					1.0
<30	1,317	24.2	1,317	24.2	
30–39	3,918	71.4	3,918	71.4	
40+	242	4.4	242	4.4	
**Maternal race/ethnicity**					1.0
Non-Hispanic Black	133	2.4	133	2.4	
Non-Hispanic White	4,380	79.8	4,380	79.8	
Hispanic	475	8.7	475	8.7	
Non-Hispanic Asian	325	5.9	325	5.9	
Other	174	3.2	174	3.2	
**Healthcare personnel**					N/A
No	–	–	2,948	53.7	
Yes	–	–	2,459	44.8	
Not reported	–	–	80	1.5	
**Urbanicity of residence**					<.01
Urban	4,657	84.9	5,080	92.6	
Rural	830	15.1	407	7.4	
**Nulliparous**					<.01
No	4,125	75.2	3,144	57.3	
Yes	1,362	24.8	2,343	42.7	
**Obesity**					<.01
No	4,032	73.5	4,422	80.6	
Yes	1,455	26.5	1,065	19.4	
**Hypertension (preexisting or HDP)**					<.01
No	4,584	83.4	4,692	85.5	
Yes	903	16.5	795	14.5	
**Diabetes Mellitus (preexisting or GDM)**					<.01
No	4,773	87.0	4,949	90.2	
Yes	714	13.0	538	9.8	
**SARS-CoV-2 infection in pregnancy**					<.01
No	3,646	66.5	5,278	96.2	
Yes	93	1.7	209	3.8	
Unknown	1,748	31.9	0	0.0	
**Plurality**					.25
Singleton	5,418	98.7	5,404	98.5	
Multiple	69	1.3	83	1.5	
**Delivery method**					.96
Vaginal	3,894	71.0	1,587	28.9	
Cesarean	1,592	29.0	3,890	70.9	
Not reported	1	0.0	10	0.2	
**Delivery year**					<.01
2019	2,605	47.5	0	0.0	
2020	2,401	37.2	12	0.2	
2021	641	11.7	5,440	99.1	
2022	200	3.6	35	0.6	
**Gestational age at delivery**					<.01
Early term (37 – 38 weeks)	840	15.3	1,622	29.6	
Term (39 – 40 weeks)	4,364	79.5	3,549	64.7	
Late or post-term (≥41 weeks)	283	5.2	316	5.8	
**Birthweight (grams)**					<.01
<2500	398	7.3	105	1.9	
2500 to 3999	4,609	84.0	4,841	88.2	
≥4000	480	8.8	463	8.4	
Not reported	0	0.0	78	1.4	
**Infant sex**					.14
Female	2,727	49.7	2,805	51.1	
Male	2,760	50.3	2,675	48.8	
Not reported	0	0.0	7	0.1	

Abbreviations: HDP, hypertensive disorder of pregnancy; GDM, gestational Diabetes Mellitus; LMP, last menstrual period; NA, not applicable.

^a^PRAMS participants include those from six states with participant report of NICU admission (Delaware, Kentucky, Mississippi, New Jersey, New Mexico, and Utah). Participants from 2021 and 2022 were excluded if they reported receiving a COVID-19 vaccine or if they did not provide a response to question asking whether they received a COVID-19 vaccine.

^b^C19VPR enrolled women who were vaccinated just prior to or during pregnancy from December 15, 2020 through June 20, 2021. Infant birth dates ranged from December 19, 2020 to February 10, 2022. C19VPR infants were restricted to those born in states with similar NICU admission rates to the included PRAMS states based on National Center for Health Statistics data (10.0 to 14.2%; Alaska, Alabama, Arkansas, Colorado, District of Columbia, Delaware, Iowa, Idaho, Illinois, Indiana, Kentucky, Louisiana, Missouri, Mississippi, Montana, North Dakota, Nebraska, New Jersey, New Mexico, Nevada, New York, Ohio, Pennsylvania, South Carolina, South Dakota, and Utah).

^c^Chi Squared tests were used to assess the difference in distributions between cohorts. Missing values were excluded from chi-squared tests assessing distribution for delivery method and infant sex. Maternal age group and race/ethnicity were matching variables; thus, distributions are completely concordant.

We obtained medical records for a convenience sample of infants with reported NICU admission (n = 148). Among these 148 infants, NICU admission was confirmed after record review for 85.1% (95% CI: 79.4%, 90.9%), suggesting that participant reports had a relatively high positive predictive value for medical record documentation.

## Discussion

This matched cohort study evaluated NICU admission as a potential concern among infants born to women receiving COVID-19 vaccines during pregnancy. The incidence of NICU admission was similar among infants born to participants who received a COVID-19 vaccine during pregnancy (C19VPR) and infants born to unvaccinated PRAMS participants. In a sensitivity analysis that matched infant pairs by state of birth, we examined confounding variables using a stepwise approach and used the most parsimonious model. While this model adjusted for different variables than the primary analysis, the results of the primary and sensitivity analyses are similar. Results are also similar in models assuming women with unknown COVID-19 illness status during pregnancy did have COVID-19 illness. No evidence of association between COVID-19 vaccination during pregnancy and NICU admission was found in any analysis.

Our results are consistent with those from previous studies, which found no increased risk of NICU admission among infants of women who received at least one dose of a COVID-19 vaccine during pregnancy compared with infants of unvaccinated women.^13–15,30–32^ Two previous studies have demonstrated a protective effect, with a lower risk of NICU admission among infants of vaccinated pregnant women.^13,15^ Lower risk of NICU admission may be attributable to lower incidence of SARS-CoV-2 infection among vaccinated mothers; in our study, fewer PRAMS participants reported known SARS CoV-2 infection during pregnancy, but status of SARS-CoV-2 infection during pregnancy was unknown for almost one-third of PRAMS participants.

Our findings add to existing evidence in two ways. First, we found no evidence of an increase in incidence of NICU admission in term-born infants whose mothers received a COVID-19 vaccine. Previous studies have included preterm infants, but few have adjusted for infant birthweight or gestational age at delivery, which are associated with NICU admission.^13–15,30–32^ An analysis assessing risk of NICU admission among full-term infants in a large registry-based study of births in Sweden and Norway demonstrated no increased risk of NICU admission among infants of women vaccinated against SARS-CoV-2 during pregnancy.^31^ Second, our study provides U.S.-based data on NICU admissions after maternal vaccination. Previous studies have been conducted in other countries, including Canada, Israel, United Kingdom, Sweden, and Norway.^13–15,30–32^ Significant regional variation in NICU admission exists, as clinical guidelines and protocols for intensive care and availability of intensive care (e.g., bed space, number of neonatal specialists, presence of transition nurseries) differ geographically, especially for infants born ≥37 weeks’ gestation.^33–35^

Previous data demonstrate overreporting of NICU admission in PRAMS, compared to medical records, with a positive predictive value of 74.6%,^36^ lower than that observed in the C19VPR cohort (85%). While we assessed positive predictive value among 148 C19VPR participants, we did not have medical records for the PRAMS participants in this study. Nearly 43% of C19VPR participants self-identified as healthcare personnel, higher than the general population; employment history was not available for PRAMS enrollees. Participant report of NICU admission likely differs by knowledge and experience with healthcare. Receipt of care from NICU staff, transfer to the NICU for a short observation period, or admission to a special care nursery may be perceived inaccurately as a NICU admission. Differences in participant employment may contribute to differences in positive predictive values, which may bias our results away from the null.

This report has several strengths, including enrollment of infants born to women vaccinated in the pre-pregnancy period (within 30 d before the last menstrual period) and first trimester, whereas many other studies did not include, or had a small percentage of, infants of women vaccinated in the first trimester.^13,30,32^ We were able to assess associations between NICU admission and timing of vaccination relative to pregnancy. We matched participants by two important potential confounders, race/ethnicity and age, improving comparability between the C19VPR and PRAMS cohorts. We documented good positive predictive value between participant-report and medical record data for NICU admission.

This report has several limitations. C19VPR does not have an unvaccinated group; therefore, we used an unvaccinated cohort from PRAMS. C19VPR and PRAMS participants may not be directly comparable for several reasons. First, C19VPR is a convenience sample of early COVID-19 vaccine adopters and subject to selection bias. Second, although PRAMS employs a random sampling strategy, states may oversample for certain characteristics (e.g., preterm births) that may result in higher NICU admissions compared to a nationally representative sample.^38^ Third, the PRAMS cohort only included participants from six states, while C19VPR participants lived primarily in the Southeastern United States. Regional and state variation in COVID-19 vaccine uptake has been documented in state immunization information systems data.^37^ Although models adjusting by region or state were unstable due to sparse data for some states, we could adjust for urban versus rural residence. Fourth, PRAMS infants were born in 2019–2022, whereas all C19VPR infants were born after December 2020. Although some studies suggest incidence of NICU admission did not change during the COVID-19 pandemic, others identified decreases in NICU admissions, especially among full-term infants, likely due to changes in thresholds for NICU admission and earlier hospital discharge to reduce virus transmission.^38–41^ Though we adjusted for birth year, changes in neonatal care practices during the COVID-19 pandemic may influence differences in NICU admission observed in this study. Data collection also differed for C19VPR and PRAMS participants. Data from PRAMS participants were collected 2–6 months after delivery whereas data from some C19VPR participants were collected as early as 4 weeks after delivery to over 6 months after delivery, depending on the timing of enrollment relative to pregnancy outcome. Because the C19VPR cohort is relatively homogenous, we were only able to match 45% of C19VPR participants with PRAMS participants. However, NICU admission incidence was not significantly different between matched and unmatched C19VPR infants. We were unable to assess timing of NICU admission after birth, length of NICU stay, and reason for admission as these variables were not collected in PRAMS. Therefore, we were unable to discern if there were any differential patterns in NICU admission and could not differentiate between NICU admissions for serious conditions and those for minor conditions that needed a brief monitoring period Lastly, we could not adjust for some potential confounders, such as some maternal chronic diseases, socioeconomic status (e.g., insurance, adequacy of prenatal care), and intrauterine substance exposure.^33,42^

## Conclusion

Our report found no evidence for an increased risk of NICU admission among full-term infants whose mothers received a COVID-19 vaccine just prior to or during pregnancy (C19VPR) compared to unvaccinated mothers (PRAMS). Our findings are consistent with those from other studies evaluating NICU admission among infants of COVID-19 vaccinated mothers and contribute to the evidence suggesting that COVID-19 vaccination during pregnancy does not increase NICU admission.

## Note

[a]. Additional states included in the analytic sample for C19VPR with NCHS NICU incidence rates greater than 10.0% and less than 14.2% included Alaska, Alabama, Arkansas, Colorado, District of Columbia, Delaware, Iowa, Idaho, Illinois, Indiana, Kentucky, Louisiana, Missouri, Mississippi, Montana, North Dakota, Nebraska, New Jersey, New Mexico, Nevada, New York, Ohio, Pennsylvania, South Carolina, South Dakota, and Utah.
